# Dignity in Medicine: Definition, Assessment and Therapy

**DOI:** 10.1007/s11920-024-01506-3

**Published:** 2024-05-29

**Authors:** Luigi Grassi, Maria Giulia Nanni, Michelle Riba, Federica Folesani

**Affiliations:** 1 Institute of Psychiatry, Department of Neuroscience and Rehabilitation, University of Ferrara, Via Fossato di Mortara 64°, 44121 Ferrara, Italy; 2 Integrated Department of Mental Health, University Hospital Psychiatry Unit, Ferrara, Italy; 3 Department of Psychiatry, and PsychOncology Program, University of Michigan Rogel Cancer Center, Ann Arbor, MI USA

**Keywords:** Dignity, Dignity-in-care, Dignity assessment, Dignity therapy, Chronic progressive disorders, Oncology, Palliative care, Psychiatry

## Abstract

**Purpose of Review:**

Over the last 20 years, dignity and dignity-conserving care have become the center of investigation, in many areas of medicine, including palliative care, oncology, neurology, geriatrics, and psychiatry. We summarized peer-reviewed literature and examined the definition, conceptualization of dignity, potential problems, and suggested interventions.

**Recent Findings:**

We performed a review utilizing several databases, including the most relevant studies in full journal articles, investigating the problems of dignity in medicine. It emerged that dignity is a multifactorial construct and that dignity-preserving care should be at the center of the health organization. Dignity should be also regularly assessed through the tools currently available in clinical practice. Among dignity intervention, besides dignity models of care, dignity intervention, such as dignity therapy (DT), life review and reminiscence therapy, have a role in maintaining both the extrinsic (preserved when health care professionals treat the patient with respect, meeting physical and emotional needs, honors the patient’s wishes, and makes attempts to maintain privacy and confidentiality) and intrinsic dignity (preserved when the patient has appropriate self-esteem, is able to exercise autonomy and has a sense of hope and meaning).

**Summary:**

Unified trends across diverse medical contexts highlight the need for a holistic, patient-centered approach in healthcare settings. Challenges compromising dignity are pervasive, underscoring the importance of interventions and systematic efforts to address these issues. Future research and interventions should prioritize the multifaceted nature of dignity, striving to create healthcare environments that foster compassion, respect, and dignity across all medical settings.

## Introduction

Dignity, as a core tenant of human life and human rights, has been studied in many different areas from philosophy to bioethics, from law to sociology, as well as medicine. In Greek, dignity was considered part of ἀρετή (arête), which is often translated as "virtue" or "excellence" and which encompassed a wider range of qualities, including moral, intellectual, and physical excellence, than dignity. The word dignity derives however from the Latin *decus* (ornament, distinction, honor, glory, but also worthiness of honor and esteem) and *dignitas,* which is “*an individual or group's sense of self-respect and self-worth, physical and psychological integrity and empowerment”* [1, 2]. There is a strong overlap between dignitas and ἀρετή, since both concepts are essential qualities for individuals to possess to live a fulfilling and meaningful life; and both emphasize the importance of treating individuals with respect, compassion, and understanding, regardless of their social status, health, or abilities.

Later, dignity has been the object of essays and reflection from Pico della Mirandola *Oration on the Dignity of Man* (*De hominis dignitate)* [3] that, as the Manifesto of the Italian Renaissance, remapped the human landscape to center all attention on human capacity and human perspective. According to Kant, dignity “*is an inviolable property of all human beings, which gives the possessor the right never to be treated simply as a means, but always at the same time as an end, because of its ultimate moral worth”* [4]. What Kant proposes is, however, a global concept, which is related to the basic dignity as a part of our beingness. Thus, it corresponds to the dimension of one’s own intrinsic worth as an inherent and inalienable value that belongs to every human being simply by virtue of being human [5]. From a different perspective, Marcel criticizing the rationalist Kantian view, situated dignity in inter-human and inter-subjective relationships and defined it as connected to the fragile vulnerable finitude of the human individual. In this it lies the ability of the individual to creatively resist attempts to humiliate him and in his effort to recognize his unique human value which contrasts with his effort to recognize his unique human values [6]. A phenomenological and ontological perspective on dignity identifies it as the “heritage of Being”, that is carrying within oneself the immensity of Being (in the ontological sense) within the limits of being human (in the ontic sense). Dignity lies in the consciousness of one’s value and limit, a conjunction of “honor and wound”, that is the intrinsic worth and vulnerability of the human condition.

It is also true that dignity has been considered a problematic area, with critics coming both from past philosophers (e.g. Schopenhauer who underscored that the idea of dignity “can be applied only in an ironical sense” and that it is “the shibboleth of all perplexed and empty-headed moralists”) and more recent skeptical scholars, who underline the fact that the concept of dignity is ambiguous (with the risk that it is incomprehensible, susceptible to abuse or to become an umbrella term for different dimensions) or it is useless, not being different from respect and personal autonomy [7, 8

However, the importance of these concepts in the medical contexts is evident, since the dimension encompassing the idea of upholding the patients’ autonomy and respecting their choices, even when they are vulnerable or incapacitated, and the need to preserve their dignity, imbue the whole health care system. With respect to this, the Declaration of Helsinki, as proposed by the World Medical Association since 1964, is based on the ethics of conducting research in the medical field underlining the respect for the individual, the right to self-determination and to make informed decision as a way to protect the individual and, in a more general sense, his or her dignity. In fact, ethics plays a role in the debate on dignity especially when controversy exists between person-defined dignity (e.g., use of tribal medicine embedded in the society values) and ethical principles (e.g., lack of support of beneficence or safety-risk issues). Besides ethical implications the themes of dignity in terms of dignity preserving care and intervention preserving dignity among patients with somatic and psychiatric disorders have concentrated the attention of psychosocial literature over the last twenty years, according to the need to change the objectifying biotechnological approach in medicine into a more dignified and subjective approach.

The aims of the present critical review are (i) to examine and discuss the most significant issues related to the area of dignity when applied in clinical care contexts; (ii) to summarize the most relevant data concerning the intervention of dignity, mainly but not only dignity therapy, among patients with somatic and/or psychiatric disorders.

## Methods

A search was made of the major databases over the last 23 years (Embase/Medline, PsycLIT, PsycINFO, the Cochrane Library) from January 2000 to December 2023, by including the most relevant studies in full journal articles investigating dignity and dignity intervention in medicine Studies published in conference proceedings, qualitative research, commentaries and discussions, letters, books, book chapters or research not published in the English language were excluded. Literature search was performed with the search terms “dignity[title] OR “dignity therapy *"[title] OR “dignity-in-care"[title] OR “dignity conserving care"[title]) AND (---* OR ---) with appropriate filters (abstract, humans, English). Full-text review of the included studies was carried out, and data were extracted on study’s characteristics and outcomes. All the disagreements were resolved with consensus amongst the authors.

## Results

The literature review allowed us to extrapolate data on several topics: the need to conceptualize dignity in medicine to have a framework of reference when working in health care settings; and the application of intervention, namely Dignity Therapy (DT) in medical areas.

### The Dimensions of Dignity in Medicine

Although the concept of dignity in medicine is complex, this notion regards the intertwining dimensions of worthiness of respect or attributable dignity, that demands affirmation, and calls for action, approval, and support from others, with the need to consider human dignity both as a status and as a value [9]. Sulmasy [10] considers dignity consisting of an *intrinsic component* (i.e. worth, stature, or value that human beings have simply because they are human) and *attributed component* (or social dignity or created dignity) (i.e. worth, stature, or value that human beings confer upon others by acts of affirmation). This perspective is shared by others when referring to dignity-of-self (i.e. the dignity we attach to ourselves as integrated and autonomous persons) and dignity-in-relation (the dignity that the individual perceives or does not perceive in the eyes of others within interpersonal relationships). In medicine, Chochinov et al. [11, 12] developed a possible structure of dignity as resulting from three primary sources (Table 1), which also defines the relationship between patients and their health care professionals [13, 14]. In a study of 9 qualitative and 13 quantitative studies, a series of components of dignity have been explicitly underlined by the patients themselves who considered this dimension as determined by the overlapping between several components (e.g., autonomy, respect, acceptance), including spiritual and faith issues [15*, 16]. Through a dignity card-sort tool (p-DCT) it has been indicated that the preservation of their dignity at the end of life could be identified as extrinsic (i.e. respect, care-tenor) and intrinsic (i.e. autonomy, self-respect, spirituality) which can be examined by clinicians as key factors in dignity-in-care [14].

**Table 1:** Components of dignity

**Chochinov Model of Dignity** [11]
• Illness-related concerns (i.e. concerns related to symptoms of physical and psychological distress, functional capacity, cognitive acuity) that threaten or impinge on the individual sense of dignity • Dignity-conserving perspectives and practices (dignity conserving repertoire) (i.e. continuity of the self, role preservation, maintenance of pride, hopefulness, autonomy/control, acceptance, resilience, living in the moment, seeking spiritual help, maintaining normality) • Social aspects of dignity (i.e. privacy boundaries, social support, care tenor, burden to others, aftermath concerns)
**Cross-cultural issues in dignity** [15]
*Extrinsic dignity* • respect • care-tenor (the attitude others demonstrate when interacting with the patient) *Intrinsic dignity* • autonomy • self-esteem • spirituality
**Patients’ definition of dignity** [16•]
• Autonomy/control (i.e., Independence, cognitive intact, medical decision making, good death) • Respect (i.e. Human being, Empathetic care Privacy and space) • Worthy self (i.e. Self identity, Continuity of self, Worthiness/value) • Family connectedness (i.e. Family support Communication and expression • Acceptance (i.e. Accept the impermanence of life, Accept approaching death, Living in the moment) • Hope/future (Hopefulness, Future planning) • God/religious (Trusting in God, Punishment from God)

There is indeed a myriad of ways in which patients’ dignity can be compromised in health care settings. These include rudeness, indifference, condescension, dismissal, disregard, intrusion, objectification, restriction, labeling, contempt, discrimination, revulsion, deprivation, and assault [17]. These can emerge in the context of asymmetrical relationships between patients and health care professionals. Also, the structure of the health care system can result in bureaucratic organizations in which workers experience increasing demands and caseloads, inadequate resources, and uncertainty about the best way to approach their work [18]. These are all obstacles to a dignified, compassionate, and humanized approach. Task-based culture prioritizes meeting targets over the provision of patient centered care; spending time filling out forms rather than attending to the psychological and spiritual dimensions of patient suffering; and models of care which value “doing” things rather than “reflecting” on the holistic nature of the patient experience [19–21].

#### Dignity at the End of Life

Dignity has been explored in a number of studies in palliative medicine, according to the central concept that maintaining personal dignity is crucial for end-of-life care and that having one’s human value and worth acknowledged; being cared for with respect and empathy; having a voice regarding one’s dying process; minimising physical and emotional suffering; safeguarding one’s privacy; maintaining emotional connection with others; resolving personal affairs, and having access to spiritual support are all within the definition of dying with dignity [22, 23]. Chochinov et al. [11] evaluated the perception of dignity and dignity-related distress in terminally ill patients, developing a model according to the main themes that emerged from qualitative interviews (Table 1). Patients displayed no difficulties in identifying the issues compromising dignity and increasing distress, and the loss of dignity was a great concern in individuals at the end-of-life increasing psychological and symptom distress [24]. The same authors [25] found that a very high percentage of patients in an advanced stage of cancer would consider spiritual and interpersonal problems as needing attention from health care professionals, such as feeling a burden to others (87.1%), feeling of not making a meaningful and/or lasting contribution in one’s own life (83.7%), and not feeling worthwhile or valued (81.4%). About 50% of patients with advanced stages of cancer indicated that overcoming their fears, finding hope, finding meaning in life, finding spiritual resources; having someone to talk to about finding peace of mind were significant needs to be responded to [26]. Also, physicians tend to have a limited vision of dignity, wherein they consider the physical aspects of suffering most influential in preserving dignity, while patients’ caregivers have a broader perspective that includes the significant role of psychosocial aspects in preserving dignity at the end of life [27].

A number of further reviews have examined the importance of dignity in palliative medicine with a general agreement about what dignity should be the essence of part of care at the end of patients’ life [28–30].

#### Dignity in Other Areas of Medicine

In other medical settings, such as cardiology, neurology, infectious disease (e.g. HIV infection), nephrology, respirology, and oncology, studies have shown that patients’ needs are not being addressed by multidisciplinary health care teams, causing an increase of suffering and non-dignity experience [31–34]. By examining patients with amyotrophic lateral sclerosis (ALS), chronic obstructive pulmonary disease (COPD), end stage renal disease (ESRD) Chochinov et al. [35] found that some aspects of dignity, (e.g., feelings of being a burden to others, uncertainty, feeling of not having control over one’s own life) differed across these conditions.

Most patients with chronic medical disorders experience problems in maintaining or regaining their sense of dignity in the face of progressive loss [36]. Three different trajectories of dignity exist and change over time: the individual's sense of dignity temporarily diminished, and the followed by a return to previous levels (Dynamic Equilibrium); the sense of dignity diminished with progression of the disease without a return to previous levels (Downward Trend); the sense of dignity remaining unaltered despite changes in circumstances (Stability).

Loss of dignity has been associated with both physical (e.g. lack of energy, pain, shortness of breath,) and psychological symptoms (e.g. anxiety, sadness, irritability) [37]. In contrast, treating patients with dignity is an independent factor related to higher satisfaction, adherence and receptivity to preventive care [38].

Individuals with severe neurological disorders, such as Motor Neuron Disorders (MND), commonly experience heightened psychological distress, including anxiety and hopelessness, alongside a decline in quality of life [39, 40]. Although this may not consistently diminish the dignity of life, it can lead to requests for hastened death [41]. In the context of Multiple Sclerosis (MS), various personal and social factors interact to either uphold or compromise dignity. The internal dialogue significantly boosts self-respect, self-awareness, and self-esteem. Patient knowledge, intertwined with societal awareness of MS, empowers patients toward greater independence.

The interplay of patients' and society's values shapes attitudes and beliefs about illness, influencing their dignity perception. Cultural and religious beliefs impact how individuals with disabilities are viewed. The restoration or maintenance of dignity in individuals with MS involves a synergistic blend of personal and social resources, encompassing education, occupation, financial status, and physical capabilities, along with external support from family, friends, relatives, and charitable sources [42].

#### Dignity in Elderly People and Early Dementia

The perception of dignity among elderly individuals, particularly those experiencing early dementia, presents a complex and multifaceted issue. Aging often coincides with a perceived decrease in societal contributions and an increase in personal needs, potentially fueling feelings of invisibility and undervaluing [43•]. This sense of invisibility can lead to concealment of symptoms and suffering, a desperate attempt to regain recognition and maintain a semblance of independence. Living under a “cloak of invisibility” could prevent the elderly to raise their voices, to feel heard and taken seriously as people. Participation in meaningful activities and maintaining societal roles emerge as crucial factors in preserving a sense of purpose and dignity for older adults. Research conducted by advocacy groups highlights the concerning disconnect between the ideal of "dignity-in-care" and the reality experienced by many elderly individuals within healthcare settings [44].

Negative interactions with staff, compromised privacy, and disregard for individual needs and preferences are identified as key factors undermining dignity in care homes and elderly health services. Additionally, the loss of autonomy and control over daily lives further contributes to feelings of disempowerment and diminished dignity [45]. Furthermore, the presence of chronic physical illnesses and the associated need for increased medication often led to diminished physical functioning and difficulties with activities of daily living.

This decline in physical abilities further threatens individual autonomy, contributing to feelings of vulnerability and potentially impacting dignity [46]. Elderly individuals with mental disorders encounter heightened challenges to their dignity due to ageism, increased risk of abuse, social stigma, and discrimination. Loneliness, dependence, and limited policy support compound their vulnerability, along with potential neurocognitive disorders, institutionalization, and healthcare inequalities [47].

#### Dignity in Psychiatric Disorders

Issues related to dignity in people with serious mental illness (SMI) disorders have been indirectly addressed under the rubric of stigma [48] and violations of human rights [49] given the experience of multiple overlapping layers of inequality and discrimination within society and the health system itself [50, 51]. There are several forms of stigma: institutional stigma (i.e. organizational policies or a culture of negative attitudes and beliefs about mental illness); public stigma (i.e. a set of negative attitudes and beliefs that motivate individuals to fear, reject, avoid, and discriminate against people with mental illness),ì; self-stigma (i.e. the internalization of public stigma and prejudices influencing an individual's self-conception with secondary feelings of shame, anger, hopelessness, or despair); and label avoidance (i.e. the choice not to pursue mental health services because patients do not want to suffer the prejudice and discrimination that the label entails) are part of stigma [52–54].

**Fig. 1:**
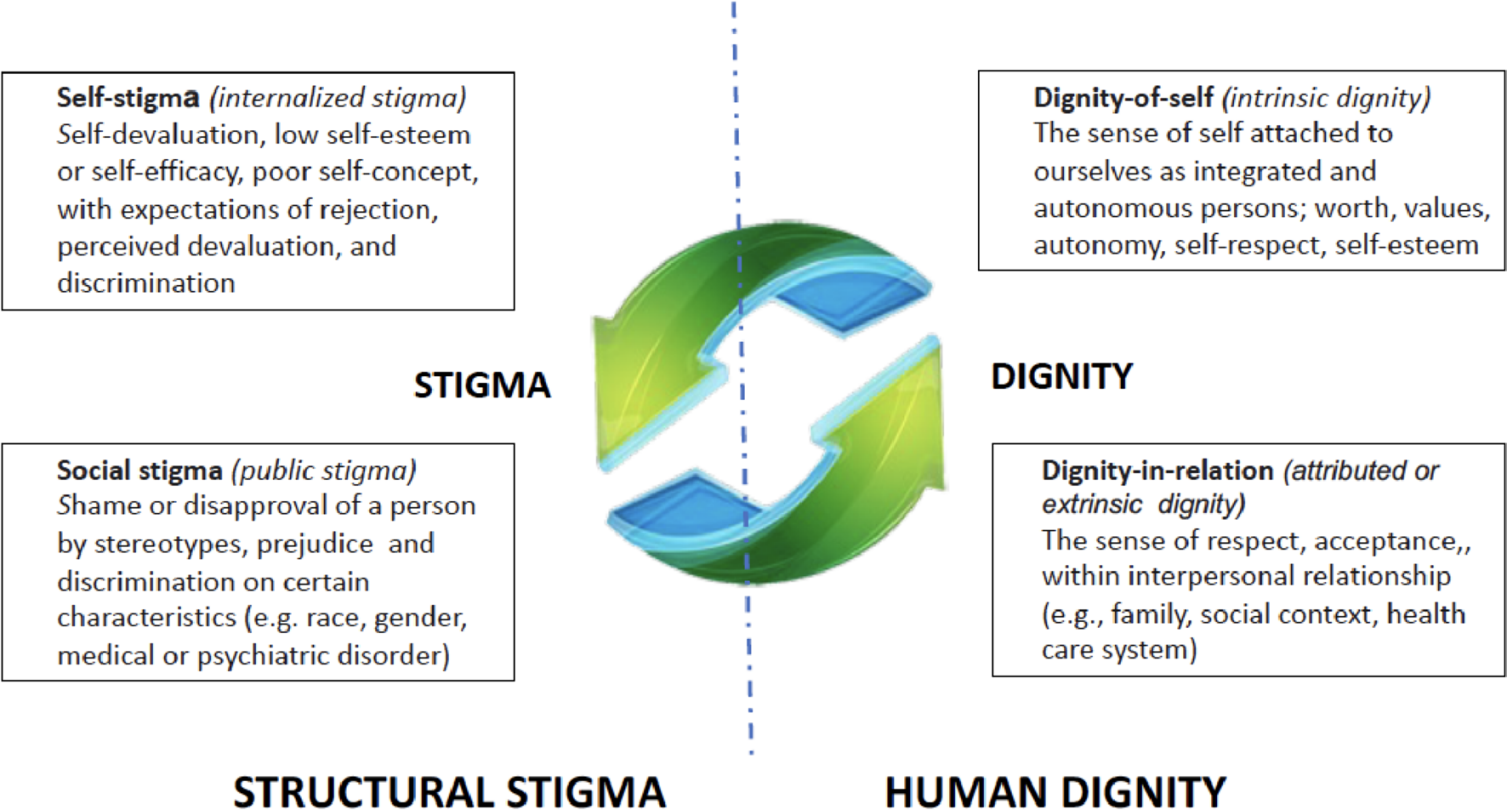
The Dignity-Stigma bipolar model as two sides of the same coin (from [57•], modified)

The literature regarding the relationship between dignity and stigma includes the notion of being treated as an equal human being, suffering related to feeling inferior, and suffering in pursuit of maintaining one's own dignity [55]. In this respect, patients with SMI perceive stigma and discrimination as omnipresent potential problems to which they remained eternally vigilant, taking various preventive behavioural and psychological measures, such as conscious and strenuous efforts to look, act and behave ‘normal’ [56]. On these bases, stigma is in fact the other side of dignity, both in terms of individual self (i.e., self-stigma vs dignity in self) and social self (i.e., social stigma vs attributed dignity), [57•] and it should be considered as a specific issue in all the medical fields and not only in psychiatry (Fig. 1).

### Assessment of Dignity in Medicine

On these data, a regular assessment of dignity is thus pivotal to preserve this vital dimension in medicine and in all the clinical context. Eliciting information regarding patient personhood, thus dignity, can be achieved in several ways, as it emerges from the literature.

The Patient Dignity Question (PDQ) [58] entails simply asking patients *“What do I need to know about you as a person to give you the best care possible?"*. In a study, 93% of palliative care professionals felt that the responses emerging from this simple question was important information for them to know, and 99% would recommend the PDQ for others. Amongst health care professionals, 90% indicated that they learned something new from the PDQ, 64% that they were emotionally affected by it, 59% that it influenced their sense of empathy, and 44% that it influenced their care [56].

In nursing homes, the application of TIME (This Is ME) [59], a short tool to address personhood, has also been proposed. TIME has been shown to influence health care professionals by favoring their attitude, care, respect, and empathy toward the residents, as well as enhanced a sense of connectedness they feel towards residents and their satisfaction in providing care.

This is similar to the WAY method (Who Are You?) which also helps in giving a different perspective on the patient as a person, rather than a body with a pathological disorder, as repeatedly reported in narrative-oriented medicine, which makes the relationship with a patient more trusty, empathic and fruitful [60, 61].

Besides dignity and person-oriented questions a series of more specific tools have been developed to assess dignity in a more specific way. A recent review [62••] explored the available instruments that directly (dignity as a concept) or indirectly (its possible components) measure dignity.

The Measurement Instrument for Dignity AMsterdam (MIDAM) [63] and its version Dignity AMsterdam-for Long-Term Care facilities (MIDAM-LTC) [64] are among such tools. The MIDAM consist of 26 items (symptoms or experiences) categorized in 4 domains, namely evaluation of self in relation to others; functional status; mental state; and care and situational aspects.

Also, the dignity card-sort tool (DC) has been developed to be used to rank factors influential in the loss or preservation of dignity at life’s end [15, 65].

A further tool is represented by the Inpatient Dignity Scale (IPDS) [66], a 35-item questionnaire with a four-factor structure: respect as a human being; respect for personal feelings and time; respect for privacy; and respect for autonomy.

According to his model [67] (Table 1), Chochinov developed the Patient Dignity Inventory (PDI), consisting of 24 items which dignity expressed, by factor analysis, five-factor sub-scales, namely symptom distress, existential distress, dependency, peace of mind, and social support. The PDI has been translated and validated in several countries, including Iran [68], Italy [69, 70], Germany [71], Greece [72], Spain [73], and the Netherlands [74]. The PDI has been validated in several setting including palliative care settings, oncology, psychiatry, geriatrics, with positive results in terms of helping health care professionals to know better the patient as a person and to highlight the most important needs related to dignity or non-dignity experiences.

The Dignity Impact is a further 7-item a scale derived from selected items in a post-test Patient Feedback Questionnaire after receiving Dignity Therapy. It has been shown to be helpful in examining the impact and the change in the individual perception of dignity after dignity-oriented treatments (e.g. DT or other interventions). A recent review of 8 articles published confirmed the need to explore the impact of dignity intervention on both the recipient and the family members [75•].

### Dignity Oriented Intervention

If dignity should be routinely part of every assessment to all patients in the several settings (e.g. outpatient and inpatient units, hospice, other facilities), [76] the next question is how to intervene in order to protect and increase it in clinical practice.

#### Dignity-in-Care Approach

A transformation from a techno- bureaucratical orientation to a more humanized, healing perspective and dignity conserving care model is the first step in this direction [11]. It is widely recognized that education in medicine should focus on an a human approach that facilitates the recognition of the patients’ personhood, by using empathy and compassion within the professional encounters [77] in all clinical settings (e.g., general medicine [78], nursing [79] and mental health care [80]). According to Chochinov [81], such an approach, defined dignity in care, can be taught, and in this way the health care system can be changed. The same author [82] and others [83–85] have examined facets of communication that are needed to achieve optimal therapeutic effectiveness, consistent with a dignity-conserving, person-centered approach which are part of the practical actions to be taken to implement basic dignity intervention (Table 2). A number of reviews and studies underscore the benefit of a dignity-in-care approach [86, 87], showing the improvement not only in the satisfaction of the patients [88] but also of the health care professionals, as well as of the healthcare outcomes [89]. Besides this approach, more specific interventions have also been developed to increase dignity, because of a person-centered medicine, including dignity therapy, life revie, and reminiscence therapy.

**Table 2:** Dignity-conserving care and communication strategies

**Skills for good and dignity oriented communication** [83, 84]
• Capacity to impart confidence (e.g. greeting patient with warmth, making eye contact, encouraging patient queries) • Empathy (e.g. eliciting patient’s concerns, acknowledging distress, recognizing and being sensitive to emotions) • “Human touch” (e.g. using appropriate physical contact, being attentive and present to the patient and the situation, being showing interest and compassion) • Relating on a personal level (e.g. asking patient about his/her life, acknowledging patient’s family, remembering details about patient’s life from visit to visit) • Being forthright (e.g. being honest and not withholding information, asking patient to recap conversation to ensure understanding) • Being respectful (e.g. listening carefully and not interrupting, taking care of the dignity of the patient) • Being thorough (e.g. providing detailed explanations, giving instructions in writing, following up in a timely manner)
**Some ingredients for therapeutic effectiveness** [89]
• Therapeutic approaches and pacing (e.g. listening attentively, encouraging client to talk about fear and distress, normalizing and validating client experience and distress) • Therapeutic presence (e.g., being respectful and nonjudgmental, being genuine and authentic, being trustworthy, being fully present, being compassionate and empathetic

#### Dignity Therapy

Dignity Therapy (DT) is one of the better-known therapeutic dignity-oriented approaches. Developed in the setting of palliative care, DT is a brief, empirically based intervention that aims to enhance the subjective experience of dignity among patients facing conditions that threaten their identity. At its core, DT offers the participant an opportunity to reflect upon crucial existential and relational issues, and to review aspects of their lives and of self that they wish to be remembered. These meaningful memories, values, words of wisdom, and special messages are documented within a final legacy document (generativity document), which can be shared and passed along to family members and beloved ones. According to the authors who developed DT, the intervention can be conducted, after proper training by several health care professionals, including physicians, psychologists, nurses, social workers and pastoral care providers.

The ultimate intent of DT is to lessen distress, promote quality of life, validate personhood. Some RCTs compared DT and other forms of intervention (e.g., Client Centered Care) and showed that DT benefited the patients by improving their quality of life and sense of dignity, with better improvement on spiritual wellbeing, depression and sadness, and higher patients’ satisfaction [90]. Since that time, DT has been applied, both through RCTs, single-arm trials and qualitative studies, in many different medical settings (Table 3).

**Table 3:** Studies on DT in various settings

**Paper (authors)**	**Country**	**Population**	**Type of study**	**Results**
**PALLIATIVE CARE**				
Chochinov et al. (2005) [91]	Canada and Australia	Terminally ill N = 100 F:M = 44:56 Age mean 63.9 ± 14.2	Cross-sectional pre-post-trial of DT	High satisfaction (91%)
Chochinov et al. (2011) [90]	Canada, Australia and USA	Palliative care N = 326 Three groups: DT (N = 108); Client Centred Care (N = 107); Standard Palliative Care (N = 111)	RCT	DT did not significantly reduced distress But DT group: more likely to find the intervention helpful, improving quality of life and sense of dignity. Higher improvement in spiritual wellbeing, higher reduction in sadness or depression and higher satisfaction with the intervention
Juliao et al. (2014) [92]	Portugal	Terminally ill inpatient in palliative care unit N = 80 Age mean 66.1 ± 12.9 Two groups: Standard Palliative Care (N = 41) F:M = 18:23 DT (N = 39) F:M = 19:20	RCT	Greater reduction in anxiety and depressive symptoms in the DT group
Hack et al. (2010) [93]	Canada and Australia	Terminally ill from inpatient palliative care at tertiary cancer center N = 50	Qualitative content analysis	Core values: ‘Family’, ‘Pleasure’, ‘Caring’, ‘A Sense of Accomplishment’, ‘True Friendship’, and ‘Rich Experience’
Tait et al. (2011) [94]	Canada	Terminal illness N = 12	Qualitative analysis	Three narrative types: evaluation; transition; legacy Patients’ responses fell into two text genres: eulogy and medical interview
Montross et al. (2011) [95]	USA	Community-based hospice N = 27 F:M = 12:15 Age mean 69 years	Qualitative analysis	Themes: autobiographical information, love experienced in life, lessons learned along the way, roles, accomplishments, character traits, unfinished business, hopes and dreams for others

**ONCOLOGY**				
Passik et al. (2004) [96]	USA DT delivered via videophone	Cancer (hospice care) N = 8 F:M = 2:6 Age mean 56.32 ± 7.65	Feasibility study Single group pre-post-trial of DT	DT perceived as helpful both for themselves and their family members. Most declared they were comfortable with the DT administration using videophone
Akechi et al. (2012) [97]	Japan	Advanced cancer N = 11 F:M = NA Age mean NA	Feasibility cross sectional study	The majority of the patients expressed improvement in their sense of dignity, wellbeing, meaning and purpose of life. DT was also considered useful for alleviating the suffering both of the patient and the family member
Johns (2013) [98]	USA	Metastatic cancer N = 7 All F Age mean 50.5 ± 14.4	Feasibility cross sectional	Most participants satisfied with the approach, however no benefits on depression, anxiety, distress and purpose in life
Houmann et al. (2014) [99]	Denmark	Incurable cancer N = 80 F:M = 48:32 Age mean 63 ± 13	Prospective (pre/post) evaluation design	T1: small increase in depression, small decrease in quality of life T2: increased sense of dignity, decrease perception to be a burden
Vergo et al. (2014) [100]	USA	Stage IV colorectal cancer N = 9 F 75% Age median 56	Feasibility longitudinal (evaluation at 1 month after DT)	All participants satisfied with the approach, most considered DT helpful to themselves and their family and having increased their sense of dignity No changes in physical symptoms No changes in anxiety, depression, sense of well-being, QoL rating, satisfaction with QoL, distress Increase in death acceptance at 1 month post-DT; change in non-life-prolonging goals of care and treatment choices over time
Hall et al. (2011) [101]	UK	Advanced cancer N = 45 F:M = 23:22 Age mean - DT group 64.91 ± 15.96 - Control group 65.30 ± 17.91	RCT 1. DT + standard care (N = 22) 2. Standard care only (control group) (N = 23) Evaluation at 1 week and 4 week post intervention	No difference in dignity-related distress Higher hope in DT vs control group at both follow-ups Participants in DT group reported more subjective benefits than control group
Hall et al. (2013) [102]	UK	Advanced cancer N = 29 F:M = 16:13 Age mean 62.21 ± 19.87	Qualitative analysis, framework approach	Main themes: “continuity of self”, “maintenance of pride”, “hopefulness”, and “care tenor”, in both groups; DT group: “generativity”, “reminiscence” and “pseudo life review”; control group “making a contribution”
Hall et al. (2013) [103]	UK	Advanced cancer N = 3 All F Age mean 40–50 years old	Case studies	All patients appreciated DT and considered it helpful
Chen et al. (2021) [104]	China	Hematologic neoplasms N = 66 F:M = 24:42 Age means 49.28 ± 10.76	RCT 1. Experimental group (N = 32): DT 30–60 min sessions two to three times a week 2. Control group (N = 34): usual care	Most of patients and family members satisfied with DT DT improved spiritual well-being, level of hope, emotional and social functioning at 1-week and 4-week follow-up
Xiao et al. (2022) [105]	China	Lung cancer (different stages) N = 120 F:M = 39:81 Age mean • Experimental group: 54.97 ± 8.86 • Control group: 57.10 ± 8.66	RCT 1. Experimental group: Family-oriented DT, three sessions (N = 60) 2. Control group: attention control (N = 60)	DT group: significantly greater reduction in existential distress and depression at week one, significantly greater improvement in spiritual well-being at week one and four of follow-up
Xiao et al. (2022) [106]	China	Lung cancer patient and family member (dyad) N = 45 dyads Patients 62.2% M Caregivers 55.6% F Age mean - Patients: 54.47 ± 8.94 - Caregivers: 48.27 ± 11.53	Qualitative analysis	Main themes: Benefits of the intervention ( Alleviating psychosocial distress; Improving personal relationship; Increase confidence in recovery); Risks of the intervention ( Triggering sad feelings); Factors to enhance successful dignity-conserving care ( Professional knowledge and skills; Facilitators' characteristics; Appropriate intervention timeline; Support from the leaders; Brief and flexible procedure; Integrated with health education); Difficulties and barriers to the delivery of dignity-conserving care ( Physical symptoms; Underrecognition of psychosocial care; Workload and time consumption)

**PSYCHIATRY**				
Solomita and Franza (2022) [107]	Italy (Avellino)	BD and MDD N = 10 F:M = 6:4 Mean age 53.88 ± 16.66 Duration of disease ≥ 3 years Stabilization phase Adequate cognitive abilities (Epitrack)	Observation study	Amelioration of perceived dignity (reduction of PDI scores), functioning (increasing of GAF scores) and symptom severity (reduction in BPRS mean scores) administer before and 15 days after DT
Avery and Baez (2012) [108]	USA	MDD F 61 years old	Case report	Coping with bereavement (loss of job) Increasing sense of dignity, purpose and meaning and will to live
Grassi et al. (2022) [109]	Italy	12 patients with oncologic diseases 12 patients with severe mental illnesses (SMI)	Qualitative study	Same themes among the two groups: "Meaning making", "Resources", "Legacy", "Dignity" “Stigma” in the SMI group “Injustice” in the cancer group
Avery and Savitz (2011) [110]	USA DT written (due to high paranoia/seriousness of disease)	Schizoaffective disorder M 55 years old	Case report	dignity therapy helped a patient with a debilitating mental illness faces a negative life event (worsening paranoia and agitation, need for continued hospitalization because of poor self-care)
Perito (2021) [111]	Italy	Addiction disorders and detention measures N = 10 (5 also psychiatric disorder) Age 21–60 years	Case series	BDI: change in the degree of depressive severity
Julião (2019) [112]	Portugal	Depressive symptoms F 54 years old	Case report	DT perceived as helpful in rediscovering meaning in life. Improvement in mood
Lubarsky and Avery (2016) [113]	USA	Anxiety disorder and alcohol use disorder M 46 years old	Case report	First documented report of using DT in a substance use disorder Patient perceived DT as life-story changing and reinforcing family relationships

**ELDERLY/EARLY DEMENTIA**				
Hall et al. (2012) [114]	London, UK	Older people in care homes N = 60 F:M = 40:20 Age mean - Control group: 81.38 ± 8.84 - DT group: 84.45 ± 8.08	RCT (29 controls, 31 interventions)	DT good acceptability and feasibility No significant difference was between the two groups in terms of effectiveness on dignity-related distress, quality of life, depression, and hope; both groups displayed a reduction in dignity-related distress at 8-week follow-up
Chochinov et al. (2012) [115]	Canada	Elderly in long-term care facilities N tot = 23 F:M = 18:5 Age means 80 years N = 12 cognitively intact N = 11 cognitively impaired	Feasibility study and qualitative analysis	Cognitively intact: lengthy and relevant responses. Main themes: death or loss, formative experiences, and disappointments and regrets Cognitively impaired: shorter and less pertinent answers. Main themes: impact of illness, personal characteristics, and important roles
Goddard et al. (2013) [116]	UK	Family members of older people in care homes N = 14 F:M = 9:5 Age mean 57 years	Qualitative analysis	Almost all felt the generative document was well received by their relative Impact on patients: Interaction; Reappraisal; Reminiscence; Factors effecting delivery of DT Impact on family: acquiring new knowledge enhanced communication; and potential role of the document during bereavement Impact on care homes: if documents were made available to carers, it could be useful in enhancing relationships with residents and therefore care delivery
Johnston et al. (2015) [117]	UK	N = 27 Of which N = 7 with Early Stage Dementia (ESD) F:M = 2:5 Age mean 78.43 N = 7 family members N = 7 stakeholder participants N = 6 focus group members	Feasibility study	DT feasible and well received by individuals with ESD Positive feedback on the participants’ reception of DT and its usefulness by other individuals involved
Johnston et al. (2016) [118]	UK	Early-Stage Dementia (ESD) F:M = 2:5 Age mean 78.43	Framework analysis (qualitative analysis)	Main themes: origin of values; essence and affirmation of self; forgiveness and resolution and existentialism/ meaning of life
Jenewein et al. (2021) [119]	Switzerland	ESD and family members N total = 108 N patients = 54 (of which N = 28 immediate group; N = 26 delayed group) F:M = 28:26 Age mean - Immediate group 81.2 ± 5.7 - Delayed group 77.8 ± 6.7 N family/friends = 54 F:M = 34:20 Age mean 70.2 ± 12.9	RCT on feasibility, acceptability, and preliminary efficacy	Good feasibility and acceptability (drop-out rate 11.1%), and high treatment satisfaction Immediate group: reduction of HADS and PDI scores at 3 months follow up; improvements in quality of life and spiritual wellbeing Delayed group: reduction of HADS but no other significant differences Combined sample: significant differences between baseline and follow-up in HADS, quality of life (physical, psychological, social, environment but not overall), PDI and FACIT-Sp-12

**NEUROLOGIC**				
Bentley et al. (2014) [120]	Australia	Motor Neuron Disease N = 29 F:M = 9:20 Age between 32 to 81 years	Cross-sectional study (one group, pre-test-post-test design)	At group level: No significant changes in hopefulness, dignity, or spirituality At individual level: DT may increase hopefulness in spiritual and religious individuals and some with more advanced disease High satisfaction (92.8%) Helpful to themselves (89.2%) Helpful to families (85.2%)
Bentley et al. (2014) [120]	Australia	Motor Neuron Disease, family caregivers N = 18	Cross sectional study (one group, pre-test-post-test design)	At group level: No significant changes in caregiver burden, anxiety, depression, and hopefulness At individual level: reduction of anxiety and depression Acceptability was mixed: N = 9 considered it helpful N = 4 considered it harmful/not helpful Participants considered DT more beneficial to the patient (N = 16)
Aoun et al. (2015) [121]	Australia	Motor Neuron Disease Patients N = 27 F:M = 9:18 Age mean 64.3 years ± 10.7 Family caregivers N = 18 F:M = 13:5 Age mean 59.9 ± 11.8 All spouses of patients	Repeated-measures design pre- and post-intervention	Patients: no significant changes in dignity related distress, quality of life, spiritual wellbeing, and hopefulness Family caregivers: no significant changes in caregiver burden, hopefulness, anxiety, and depression Patients displayed high levels of acceptability considering DT helpful to themselves (88.9%) and their family (81.5%) and expressed satisfaction with the intervention (92.6%), feeling it helped them feel closer to their loved ones and gave them a sense of looking after unfinished business Most of family members considered DT helpful to themselves (88.9%) and reported the importance of the generativity document as a source of comfort both in the present and the future

**OTHER**				
Testoni et al. (2020) [122]	Italy	Life convicts N = 10 All M	Qualitative study using thematic analysis	Two main themes: 1. Values of freedom, self-consciousness, and education – and their failure in prison 2. Life sentence as annihilation of life meaning and of the values of generativity and family

Numerous reviews and metanalysis of RCTs are now available and confirm the effectiveness of DT in improving the psychological well-being of patients and enhancing their quality of life [123–128]. Also, DT has been evaluated regarding the impact on the family members, with benefits underscored in terms of comfort given to the family and improvement of the process of bereavement [75, 129, 130]*. In a study [131], DT recipients had a more significant increase generativity and ego-integrity scores than life review (LR) intervention, with no significant changes for dignity-related distress or physical, social, emotional, and functional wellbeing among the groups. High acceptability and satisfaction with interventions were noted for recipients of both DT and LR and family/carers of DT participants.

Recent metanalyses, however report contradictory results. In two metanalyses, the first examining 16 studies with 1202 participants [132••] and a second involving 8 studies with 776 participants [133], significant differences were observed between DT and control groups in dignity-related distress, hope, and quality of life, but not in depression, anxiety and spiritual well-being. A different metanalysis [134••] of 9 RCTs with a total of 871 participants showed that DT did not improve terminally ill patients' sense of dignity, hope, spiritual well-being, and quality of life, while significant reduction were found in anxiety and depression after intervention.

Although DT has been developed in palliative medicine, its application has been gradually extended to other settings, such as end-stage diseases, chronic life-threating conditions, and dementia. In the latter, the role of DT can be considered as a way to alleviate suffering, and give meaning and purpose to life [135•].

DT has also been applied in mental health settings, particularly among individuals with SMI. Grassi et al. [109] compared the narratives of DT between a group of 12 patients with cancer and 12 patients with SMI and found similar categories of dignity (i.e. "Meaning making", "Resources", "Legacy", "Dignity") among the two groups, with only “Stigma” specifically emerging from people with SMI, and “Injustice”, in patients with cancer.

Finally, DT has been applied in settings where the concept of end and limit of existence is related to the social condition of being imprisoned. An Italian study [122] of a small sample of 10 male prisoners with a life-sentence were interviewed using DT and a thematic analysis was carried out to explore the main themes emerging from the interviews (i.e., values of freedom, self-consciousness and education, and the lack of their valorization in the prison context; life sentence as shattering the possibility of a life meaning, and of the values of generativity and family).

#### Other Interventions

Further interventions have been developed with the aim to increase the sense of respect and dignity of patients. Many, within the area of narrative medicine, include reminiscence and life reviews which have some connections with DT and have been extensively used especially in elderly settings [136] and palliative care [137] since many years [138, 139]. In general, it is demonstrated that memories can create positive life narratives that support mental wellbeing and growth and other psychological outcomes, [140] with application especially in the elderly and in people with dementia.

With some similarities with DT, reminiscence intervention, both in a structured or unstructured way, and on individual or group basis, conducted by different health care professionals (e.g., by nurses, social workers) consists in helping the person to think about one's life and recall memorable and pleasant events from the past (not recent or current events). Photographs or memory boxes, as well as music and music intervention, can also be used as a facilitator o the reminiscence process [141]. Data indicate that the reminiscence therapy can improve the quality of life, life adaptation, and reduce depression.

Life review and life review therapy take reminiscence to a deeper level and it is conducted by more trained therapists, including psychiatrists and psychotherapists. Participants are helped to examine both positive and negative life experiences, reconstructing them in chronological order, looking for meaning in those events and integrating them in a coherent life story and a meaningful whole. In this way, patients may resolve conflicts and complete life tasks with benefit in terms of peace of mind and meaning, when facing death [142]. A recent metanalysis, indicates that reminiscence therapy has been shown to help cancer patients to improve their quality of life and symptoms, including anxiety and depression [143••], while life review intervention may improve the meaning of life domain of spiritual well-being general distress overall quality of life [137, 144].

Recently, a life review intervention model has been developed in oncology settings to increase dignity among cancer patients. It consists of a dialogue conducted by trained therapists with the aims to discuss life events, to identify the significant elements in patients' life course, and to explore how the diagnosis has changed their values [145]. Emphasis is given to the patient's resources changes in relationships with significant others with the aim for patients to discover their potential, to identify strategies for coping with the events, to achieve a better understanding of themselves, and to discuss ultimate life goals or projects. The preliminary results on 41 patients indicate that the intervention was perceived favorably by all the participants. A change in the nurse-patient relationship was also noted and it was deemed to be beneficial [146]. Further studies exploring the impact of Life Review Therapy (LRT) and Memory Specificity Training (MST) (LRT-MST) were found to improve ego-integrity and despair among cancer patients in palliative care [147], with qualitative analysis indicating the benefit and acceptance of the intervention among the patients [148], but no specific effect on the family caregivers [148, 149].

## Discussion

This review illuminates the challenges that individuals face in preserving their sense of dignity across various disorders, underscoring the imperative adoption of a dignity-oriented care approach in medical contexts. The significance of assessing dignity-related distress and implementing effective interventions, notably Dignity Therapy (DT) and other forms of intervention based on reminiscence of one’s own existential trajectory and life-review, become apparent. DT has demonstrated transformative potential not only in palliative and cancer care but also in chronic disorders. The numerous studies on the application of DT in different settings shed light on the versatility and effectiveness of DT in settings other than the end-of-life and oncologic care [39].

The escalating prevalence of chronic diseases, marked by poor health status, symptom burden, functional disability, and cognitive impairments, necessitates a shift toward dignity-centered care [150]. The feasibility and necessity of such an approach in chronic disorders are underscored, focusing on enhancing self-esteem, alleviating multidimensional distress, and aiding individuals in finding meaning and purpose while maintaining or improving their quality of life [151]. These issues, together with living with uncertainty of illness and multilayered distress, constitute the premises to offer dignity-centered care to individuals with chronic disorders as well.

Thus, it is fundamental to assess dignity and dignity-related distress, with tools (e.g., the PDI) facilitating a person-centered care. A dignity-in-care approach is crucial for transforming healthcare systems towards a more humanized and healing perspective. Education in medicine should emphasize empathy, compassion, and communication skills to achieve optimal therapeutic effectiveness and dignity-conserving care [81]. In fact, dignity-centered care should be applied in all clinical settings disorders since the aim of improving self-esteem; relieve patients from multi-faceted distress (physical and psychological), and aid the individual in finding meaning and purpose in life are is mandatory to improve quality of life [151].

However, in chronic disorders there are challenges that necessitate to specifically focus on adaptive coping strategies, such as reorienting goals and achievements, and emphasizing remaining capabilities in a more dynamic way. Individuals with permanent disability described the main strategies they adopted to preserve their sense of autonomy, integrity, influence and participation in their daily lives, that is factors that could aid in maintaining their sense of dignity (e.g., trying to keep a private sphere; striving to communicate; searching for possibilities) [152].

Specific interventions targeting dignity-related distress have demonstrated the potential to restore quality of life and perceived dignity across various diseases, providing benefits not only to patients but also to caregivers. The review advocates for a holistic, patient-centered approach in healthcare settings, emphasizing the need for systematic efforts to address challenges compromising dignity. The ethical imperative for the diffusion of dignity-centered care across all medical settings is underscored, recognizing the vital role of healthcare professionals in nurturing compassion, respect, and dignity in patient care [153, 154].

### Limitations and Conclusions

There are a number of limitations of this review. A first regards the fact that we decided to limit our analysis on studies specifically speaking about dignity and/or measuring it as a specific outcome, leaving out gorm the studies all those indirectly having an impact on patients’ dignity. A further limitations is that we limited our research to narrative and meaning-centered intervention, specifically DT, reminiscence and life review intervention, while other forms of psychotherapeutic intervention might have been examined.

Despite these limitations, what emerged from this revie is that unified trends across diverse medical contexts highlight the need for a holistic, patient-centered approach in healthcare settings. Challenges compromising dignity are pervasive, underscoring the importance of interventions like Dignity Therapy and systematic efforts to address these issues.

Future research and interventions should prioritize the multifaceted nature of dignity, striving to create healthcare environments that foster compassion, respect, and dignity across all the contexts of care, including the challenges given by the increase of the number of immigrants, refugees from war zones and people of different cultures who are encountered with significant risk for their dignity in various medical settings.
